# Epidemiological characterization of uveitis in the elderly population: a systematic review and meta-analysis

**DOI:** 10.1007/s10792-026-04170-z

**Published:** 2026-07-19

**Authors:** Tom Liba, Eran Levanon, Alon Gorenshtein, Jacob Megreli, Gil Ben-David, Itamar Gothelf, Asaf Bar, Rita Ehrlich

**Affiliations:** 1 Azrieli Faculty of Medicine, Bar-Ilan University, Safed, Israel; 2 Faculty of Health Sciences, Goldman Medical School, Ben-Gurion University of the Negev, Beer-Sheva, Israel; 3 Ophthalmology Department, Sheba Medical Center, Ramat Gan, Israel; 4 Department of Neurology, Beth Israel Deaconess Medical Center, Harvard Medical School, Boston, USA; 5 Ophthalmology Department, Rabin Medical Center, Petah Tikva, Israel; 6 Ophthalmology Department, Wolfson Medical Center, Tel Aviv University, 62 Halohamim St, 5822012 Holon, Israel; 7 Faculty of Medical & Health Sciences, Tel Aviv University, Tel Aviv-Yafo, Israel

**Keywords:** Uveitis, Elderly, Epidemiology, Etiology, Meta-analysis

## Abstract

**Purpose:**

This systematic review and meta-analysis aimed to characterize etiological patterns of uveitis in individuals aged 60 years or older.

**Methods:**

Following the PRISMA 2020 and MOOSE reporting guidelines, we searched PubMed/MEDLINE, Scopus, PubMed Central (PMC), and Web of Science (January 1, 2005–January 1, 2025). Data extraction focused on study characteristics, sample size, uveitis location, and reported causes. Random-effects models were used to pool proportions across studies, heterogeneity assessed by the I^2^ statistic. Methodological quality was evaluated via the Newcastle–Ottawa Scale for cohort studies and the JBI Critical Appraisal Checklist for cross-sectional studies.

**Results:**

Out of 10,829 retrieved citations, 16 studies met the inclusion criteria. Idiopathic uveitis constituted the largest proportion, with a pooled proportion of 41.45% (95% CI 30.63–53.16%). Infectious etiologies included herpetic uveitis (pooled proportion 11.40%, 95% CI 7.45–17.04%), cytomegalovirus (3.09%, 95% CI 1.01–9.06%) and tuberculosis (5.10%, 95% CI 2.58–9.83%). Non-infectious causes, such as sarcoidosis (5.21%, 95% CI 2.78–9.55%) and HLA-B27-associated uveitis (6.05%, 95% CI 3.35–10.71%), also contributed substantially. High heterogeneity (I^2^ > 75%) was noted across most etiologies, likely reflecting variable study populations, diagnostic criteria, and geographic factors.

**Conclusion:**

Our meta-analysis underscores the diverse etiological landscape of uveitis in the elderly, with over 40% of cases remaining idiopathic. High heterogeneity highlights regional variations and diagnostic challenges. Standardized evaluation protocols and improved access to advanced investigations may help reduce the burden of undiagnosed cases. Within Asia, the only continent contributing multiple studies, subgroup analysis reduced heterogeneity for some etiologies, suggesting that regional factors contribute to the observed variability.

**Supplementary Information:**

The online version contains supplementary material available at 10.1007/s10792-026-04170-z.

## Introduction

Uveitis is an inflammatory condition primarily involving the uveal tract (iris, ciliary body, choroid), classified into anterior, intermediate, posterior, and panuveitis. [1]. In developed countries, uveitis is a significant contributor to vision loss [2]. Age significantly influences the presentation characteristics of uveitis, and with the global population aging, the proportion of elderly individuals is expected to increase in the coming years [3]. The World Population Aging Report (2009) defines elderly individuals as those aged 60 years or older [4]. The global population aged 65 and over is expected to grow rapidly, outpacing overall population growth in the coming decades. [5]. Various studies highlight differing common diagnoses for uveitis in elderly patients, with some identifying herpes, others sarcoidosis, and tuberculosis as the most prevalent. [6–9]. With the global population aging, the number of elderly patients with uveitis is expected to rise. This review aims to summarize the main causes of uveitis in elderly patients and highlight common patterns and gaps in current data.

## Methods

This meta-analysis and systematic review are reported according to both the PRISMA 2020 (Preferred Reporting Items for Systematic Reviews and Meta-Analyses) [10] and MOOSE (Meta-analysis of Observational Studies in Epidemiology) reporting guidelines [11]. Completed checklists for both are provided in the Supplementary Material. The study protocol was preregistered on the International Prospective Register of Systematic Reviews (PROSPERO; Registration number: CRD42024623935).

## Search strategy

The authors conducted an electronic database search of PubMed/MEDLINE, Scopus, PubMed Central (PMC), and Web of Science. The following search terms were used: Uveitis OR “Intraocular inflammation” OR “Ocular inflammation” AND Epidemiology OR Etiology OR “Clinical Patterns” OR “Clinical features” OR Prevalence OR Incidence OR “Patterns of” AND Elderly OR Geriatric OR Aged OR “Older Adults.” The full search strategies are provided in the Supplementary Material. Our search period was from January 1, 2005, to January 1, 2025. We limited our search to studies from January 1, 2005, to January 1, 2025 to capture the most recent data and reflect current diagnostic practices, following the introduction of the SUN criteria in 2005 [12]. Relevant articles were cross-referenced for additional manuscripts that were not directly found through the above search. PMC was searched as a full-text repository to complement the indexed records retrieved from PubMed/MEDLINE. No language restrictions were applied.

## Inclusion and exclusion criteria

Articles were included for analysis if they fulfilled the following criteria: (1) articles published between January 1, 2005, to January 1, 2025, (2) study period was between January 1, 2005, to January 1, 2025 (3) patients aged 60 or older, (4) cross-sectional studies or cohort studies,with a  known etiology of uveitis.

The exclusion criteria were as follows: (1) duplicate reports and/or publications of the same data. (2) outcomes that were evaluated solely through self-reported assessments.

## Data extraction and quality assessment

Three independent reviewers, T.L, A.G and J.M independently conducted title, abstract, and full-text screening, as well as data extraction, based on the inclusion and exclusion criteria above. In cases where two reviewers agreed and one disagreed, a fourth reviewer evaluated the article to make the final decision. Each study in the final analysis was independently reviewed by the authors and the following data were extracted: the first author’s name, year of publication, study period, study location, study type, sample size, data collection methods, anatomic location of inflammation (anterior, intermediate, posterior and panuveitis), etiology of uveitis, demography and other factors including age. Cases labeled as “idiopathic,” including idiopathic anterior, intermediate, posterior, or panuveitis, were grouped into a single “idiopathic” category. We defined “idiopathic” as uveitis with a complete work-up and no identified cause. “Undetermined” referred to cases with incomplete evaluation or insufficient follow-up. When terms like “unclassified” were used, we reviewed the methods section of each study. If context suggested incomplete assessment, we reclassified them as “undetermined” [8, 13]. Sensitivity analysis was done for Behçet’s disease and VKH, where multiple studies used consistent diagnostic criteria. These two conditions were selected because a sufficient number of included studies explicitly applied standardized diagnostic frameworks, the International Study Group criteria for Behçet disease and the revised diagnostic criteria for VKH disease, enabling meaningful comparison of pooled estimates when restricted to studies with consistent case definitions. During the quality assessment phase, two independent reviewers, T.L, A.G, evaluated the studies based on several criteria: whether diagnoses were made by uveitis specialists, applied clear clinical or laboratory definitions for etiology, and conducted comprehensive systemic evaluations when needed. In instances where disagreements occurred between the reviewers during this phase, a third reviewer evaluated the article to make the final decision.

## Risk of bias assessment

Cohort study quality was evaluated using the Newcastle–Ottawa Scale (NOS), Methodological quality and risk of bias were independently assessed by two reviewers, T.L, A.G, according to the NOS, which is valid for cohort studies [14]. In cases of disagreement, a third reviewer evaluated the study to make the final decision. The scale consists of three quality parameters: (i) selection, (ii) comparability, and (iii) outcome. The quality of the studies (poor, fair, and good) was scored by allocating stars to each domain as follows: a poor-quality score was allocated 0 or 1 star(s) in the selection, 0 stars in comparability, and 0 or 1 star(s) in the outcome domain; a fair quality score was awarded, two stars in the selection, one or two stars in comparability, and two or three stars in outcomes. A good quality score was awarded, with three or four stars in selection, one or two in comparability, and two or three stars in outcomes [14]. We assessed cross-sectional studies using the Joanna Briggs Institute (JBI) Critical Appraisal Checklist [15], which is suited for descriptive designs. As our meta-analysis focused on a single population group (age ≥ 60) without between-group comparisons, the Newcastle–Ottawa Scale adaptation was not appropriate, The “Comparability” domain would have incorrectly categorized high-quality studies as low quality [16]. The questionnaire contains eight questions that were answered with yes, no, or unclear. A score of “yes” for > 5 times, 3–4 times, and 0–2 times are considered high methodological quality, moderate methodological quality, and low methodological quality, respectively [15].

## Data tabulation and statistical analyses

The primary effect size was the proportion of each uveitis etiology among all elderly uveitis cases within each study. Extracted data were tabulated into standardized spreadsheets recording study characteristics, sample sizes, and the number of cases attributed to each etiology, as detailed in Tables 1 and 2. The effect size analyzed was the proportion and its 95% confidence intervals (95% CIs) for each study. A random-effects model was used to pool proportions across studies, employing the restricted maximum-likelihood estimator (REML) to estimate between-study variance (τ2). Proportions were transformed using the logit transformation prior to pooling. The Hartung-Knapp adjustment was applied for the confidence interval of the pooled effect. The results were pooled when the number of studies was ≥ 2, and a forest plot was created to display the data. For each etiology, only studies that explicitly reported case counts for that condition were included in the corresponding meta-analysis; studies that did not report a given etiology were not assumed to have zero cases, as the absence of reporting may reflect differences in classification systems rather than true absence. When a study explicitly reported zero cases for an etiology, that zero-event entry was included. Etiologies with fewer than five contributing studies should be interpreted with particular caution, as the between-study variance may be imprecisely estimated. Heterogeneity between studies was assessed using Cochran’s Q test, with the null hypothesis that all studies share a common true proportion (i.e., no between-study heterogeneity). I2 was used to quantify the proportion of total variability attributable to between-study heterogeneity rather than sampling error, with values above 50% indicating substantial heterogeneity. Values of p < 0.05 were considered statistically significant. Publication bias was assessed using funnel plots when more than 10 studies were available for a given outcome. Funnel plots of the standard error by the transformed proportion were generated for outcomes reported by more than 10 studies. Funnel plot asymmetry was assessed using Egger’s regression test, with the null hypothesis that the regression intercept equals zero, applied to the transformed proportions and their standard errors to detect small-study effects. Continental subgroup analyses were performed for etiologies with I2 exceeding 75% to explore geographic sources of heterogeneity. Leave-one-out sensitivity analyses were conducted for de la Torre et al., the largest contributing study, to assess its influence on pooled estimates. Additionally, sensitivity analyses were conducted for Behçet disease and Vogt-Koyanagi-Harada disease by restricting the analysis to studies that explicitly applied standardized diagnostic criteria (International Study Group criteria for Behçet disease [17]; revised diagnostic criteria for VKH disease [18]). Each study’s Methods section was reviewed to determine whether these criteria were stated. The pooled proportion from the restricted subset was compared with the estimate from all contributing studies. Data analyses were performed using R Foundation Statistical Software (version 4.4.2) with the meta package (version 8.3–0).

**Table 1:** Characteristics of the included studies

References	Country	Publish date	Study duration	Study type	Study site	Elderly sample size	Infectious versus non-infectious	Most common elderly uveitis etiologies (3)
de la Torre et al. [19]	Colombia	6/3/2024	2010–2022	Retrospective cross-sectional	Tertiary centers	837	N/A	Idiopathic- 315 (37.6), Toxoplasmosis- 126 (15.1), Virus-associated- 77 (9.2)
Shirahama et al. [7]	Japan	4/5/2021	2013–2018	Retrospective cross-sectional	Tertiary center	290	N/A	Sarcoidosis-67 (23.1), Intraocular lymphoma- 48 (16.6), CMV-47 (16.2)
Keorochana et al. [20]	Thailand	22/04/2019	2013–2018	Retrospective cross-sectional	Tertiary center	110	45:17	Herpetic- 19 (17.2), Cytomegalovirus-13 (11.8), HLA-B27- 11 (9.9)
Abaño et al. [8]	Philippines	30/10/2017	2010–2015	Retrospective cross-sectional	Tertiary center	88	20:14	Tuberculosis-10 (11.36), Toxoplasmosis-6 (6.82), Sympathetic Ophthalmia- 6 (6.82)
Dogra et al. [21]	India	13/12/2016	2011–2014	Retrospective cross-sectional	Tertiary center	136	64:16	Tuberculosis -22 (16.5), Endophthalmitis-9 (6.8), Herpetic uveitis-9 (6.8)
Zagora et al. [13]	Australia	20/11/2016	2009–2015	Retrospective cohort	Tertiary centers	327	83:121	HLA-B27 + -44 (13.4), Sarcoidosis-29 (8.9), VZV- 22 (6.7)
Amin et al. [22]	Egypt	26/10/2016	2013–2016	Retrospective cohort	Tertiary center	42	18:18	Herpes-12 (28.6), Tuberculosis -4 (9.5), Sarcoidosis -4 (9.5)
Sabhapandit et al. [23]	India	25/10/2016	2014	Retrospective cohort	Tertiary centers	107	25:10	Tuberculosis -23 (21.5), FHC- 4 (3.7), Rheumatoid arthritis- 2 (1.8)
Nguyen et al. [24]	Vietnam	11/10/2016	2011–2015	Retrospective cohort	Tertiary centers	27	3:12	VKH- 4 (14.8), Sympathetic ophthalmia-3 (11.1), Herpetic anterior uveitis- 2 (7.4)
Sukavatcharin et al. [25]	Thailand	7/9/2016	2014–2015	Prospective cross-sectional	Tertiary center	152	96:43	Cytomegalovirus -45 (29.6), Herpetic- 39 (25.7), VKH- 17 (11.2)
Lee et al. [26]	Korea	19/08/2016	2014	Retrospective cross-sectional	Tertiary centers	113	30:12	Endophthalmitis- 14 (12.3), Behçet disease- 5 (4.4), Syphilis- 5 (4.4)
Manandhar et al. [27]	Nepal	19/05/2016	2018–2018	Retrospective cross-sectional	Tertiary center	139	33:14	Herpetic- 19 (13.7), Ankylosing spondylitis- 8 (5.8), Tuberculosis-5 (3.6)
Gao et al. [28]	China	12/4/2016	2014–2015	Retrospective cohort	Tertiary centers	25	1:16	VKH- 5 (20), Sympathetic ophthalmia- 3 (12), PIOL- 3 (12)
Nakahara et al. [29]	Japan	8/3/2016	2010–2012	Retrospective cross-sectional	Tertiary center	312	82:95	Sarcoidosis- 34 (10.9), Scleritis- 32 (10.3), Herpetic iridocyclitis- 26 (8.3)
Grajewski et al. [30]	Germany	2/3/2015	2012–2013	Retrospective cohort	Tertiary center	85	24:20	Sarcoidosis- 17 (20), Herpetic- 12 (14),MFC- 4 (5)
Abdulaal et al. [6]	Lebanon	5/6/2014	2009–2011	Retrospective cross-sectional	Tertiary center	18	9:04	Herpes- 5 (27.7), Tuberculosis -3 (16.6), Sarcoidosis-2 (11.1)

VKH, Vogt-Koyanagi-Harada disease; HLA-B27 + , Human leukocyte antigen B27 positive; FHC, Fuchs heterochromic cyclitis; PIOL, Primary intraocular lymphoma; MFC, Multifocal choroiditis; Herpetic, Herpetic uveitis (general); VZV, Varicella zoster virus

**Table 2:** Pooled etiologic proportions of uveitis in the elderly population

Condition	No. of studies	Sample size	Crude proportion n/N (%)	Proportion (95% CI)	p-value	Heterogeneity (I^2^%)
Infection						
Herpetic uveitis	[15]	2563	271 (10.57%)	0.1140 [0.0745; 0.1704]	< 0.0001	85.0%
Tuberculosis	[15]	2408	103 (4.28%)	0.0510 [0.0258; 0.0983]	< 0.0001	89.3%
Cytomegalovirus	[12]	2480	123 (4.96%)	0.0309 [0.0101; 0.0906]	< 0.0001	92.6%
Toxoplasmosis	[9]	1958	152 (7.76%)	0.0305 [0.0142; 0.0643]	< 0.0001	89.8%
Syphilis	[8]	1718	30 (1.75%)	0.0210 [0.0127; 0.0344]	0.2395	23.8%
Endophthalmitis	[8]	2013	58 (2.88%)	0.0300 [0.0123; 0.0713]	< 0.0001	85.2%
ARN	[5]	602	8 (1.33%)	0.0203 [0.0033; 0.1146]	0.0148	67.7%
Non-Infection						
VKH disease	[19]	2790	84 (3.01%)	0.0336 [0.0173; 0.0643]	< 0.0001	82.5%
Sarcoidosis	[19]	2720	191 (7.02%)	0.0521 [0.0278; 0.0955]	< 0.0001	90.2%
HLA-B27 +	[9]	1821	110 (6.04%)	0.0605 [0.0335; 0.1071]	< 0.0001	82.1%
Sympathetic ophthalmia	[9]	1844	29 (1.57%)	0.0241 [0.0083; 0.0679]	< 0.0001	76.9%
Behçet disease	[10]	2154	20 (0.93%)	0.0156 [0.0055; 0.0436]	0.0006	69.2%
FUS	[7]	1834	31 (1.69%)	0.0196 [0.0121; 0.0314]	0.3060	16.2%
PSS	[7]	1853	24 (1.3%)	0.0166 [0.0069; 0.0394]	0.0216	59.5%
Rheumatoid arthritis	[6]	1530	18 (1.18%)	0.0126 [0.0080; 0.0198]	0.7200	0.00%
Choroiditis	[4]	860	26 (3.02%)	0.0341 [0.0059; 0.1730]	0.0290	66.7%
Lymphoma	[6]	1901	78 (4.1%)	0.0366 [0.0063; 0.1860]	< 0.0001	92.3%
Birdshot chorioretinopathy	[3]	1249	6 (0.48%)	0.0070 [0.0004; 0.1118]	0.0702	62.3%
Lens-induced uveitis	[5]	1394	34 (2.44%)	0.0248 [0.0143; 0.0429]	0.4050	0.2%
Undetermined	[1]	837	50 (5.97%)	Single study, not pooled†	–	–
Idiopathic uveitis	[19]	2518	1048 (41.62%)	0.4145 [0.3063; 0.5316]	< 0.0001	91.4%

VKH, Vogt-Koyanagi-Harada disease; HLA-B27 + , Human leukocyte antigen B27 positive; FUS, Fuchs’ uveitis syndrome; PSS, Posner-Schlossman syndrome

## Results

**Fig. 1:**
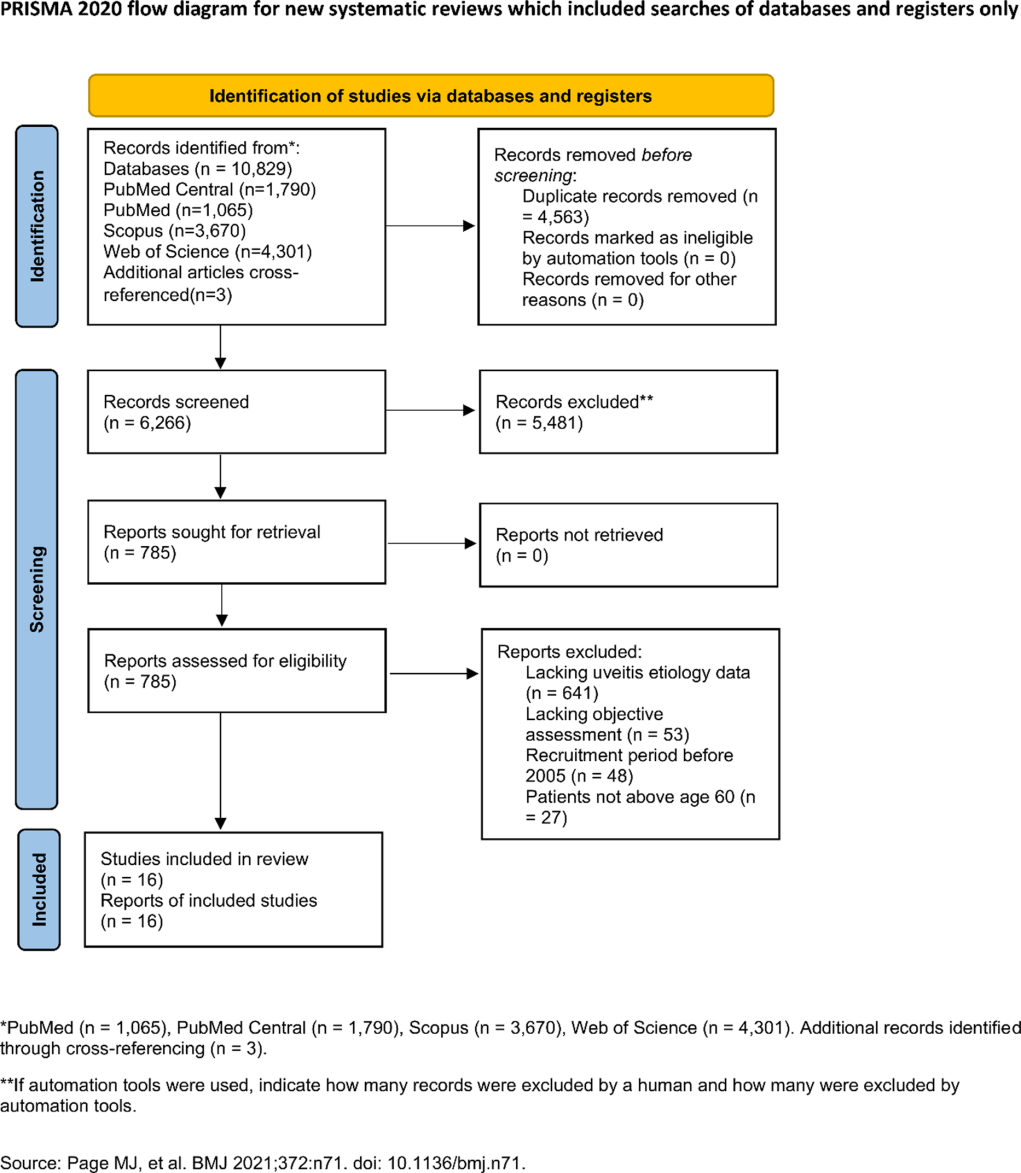
PRISMA 2020 flow diagram for selection of articles

A comprehensive literature search yielded 10,829 studies from databases. After removing 4563 duplicates, 6266 studies were screened for relevance based on titles and abstracts. Of these, 785 studies were evaluated for full-text eligibility. Following exclusions due to insufficient etiology data, inappropriate study populations, or lack of objective assessment, 16 studies were included in the systematic review and meta-analysis. The PRISMA flow diagram details the study selection process (Fig. 1).

Table 1 summarizes the characteristics of the included studies. The 16 included studies spanned 13 countries across Asia (12 studies), Oceania (1), Europe (1), Africa (1), and South America (1). Sample sizes ranged from 18 to 837 elderly uveitis patients (total N = 2,808). All studies were conducted at tertiary referral centers. Publication dates ranged from 2014 to 2024, with study recruitment periods covering 2009 to 2022.

Table 2 details the meta-analysis results of uveitis proportions by etiology. Idiopathic uveitis accounted for the largest proportion, with a pooled proportion of 41.45% (95% CI 30.63–53.16%). Among infectious etiologies, herpetic uveitis had a pooled proportion of 11.40% (95% CI 7.45–17.04%), tuberculosis 5.10% (95% CI 2.58–9.83%), cytomegalovirus 3.09% (95% CI 1.01–9.06%), and toxoplasmosis 3.05% (95% CI 1.42–6.43%). Among non-infectious etiologies, HLA-B27-associated uveitis had a pooled proportion of 6.05% (95% CI 3.35–10.71%) sarcoidosis 5.21% (95% CI 2.78–9.55%), and lens-induced uveitis 2.48% (95% CI 1.43–4.29%). Heterogeneity between studies was substantial for most etiologies, with I2 values exceeding 75%.

Despite high heterogeneity, pooling was considered appropriate given the descriptive nature of the analysis and the use of random-effects models, which incorporate between-study variance. The pooled estimates should therefore be interpreted as average proportions across heterogeneous clinical settings rather than precise universal figures.

**Fig. 2:**
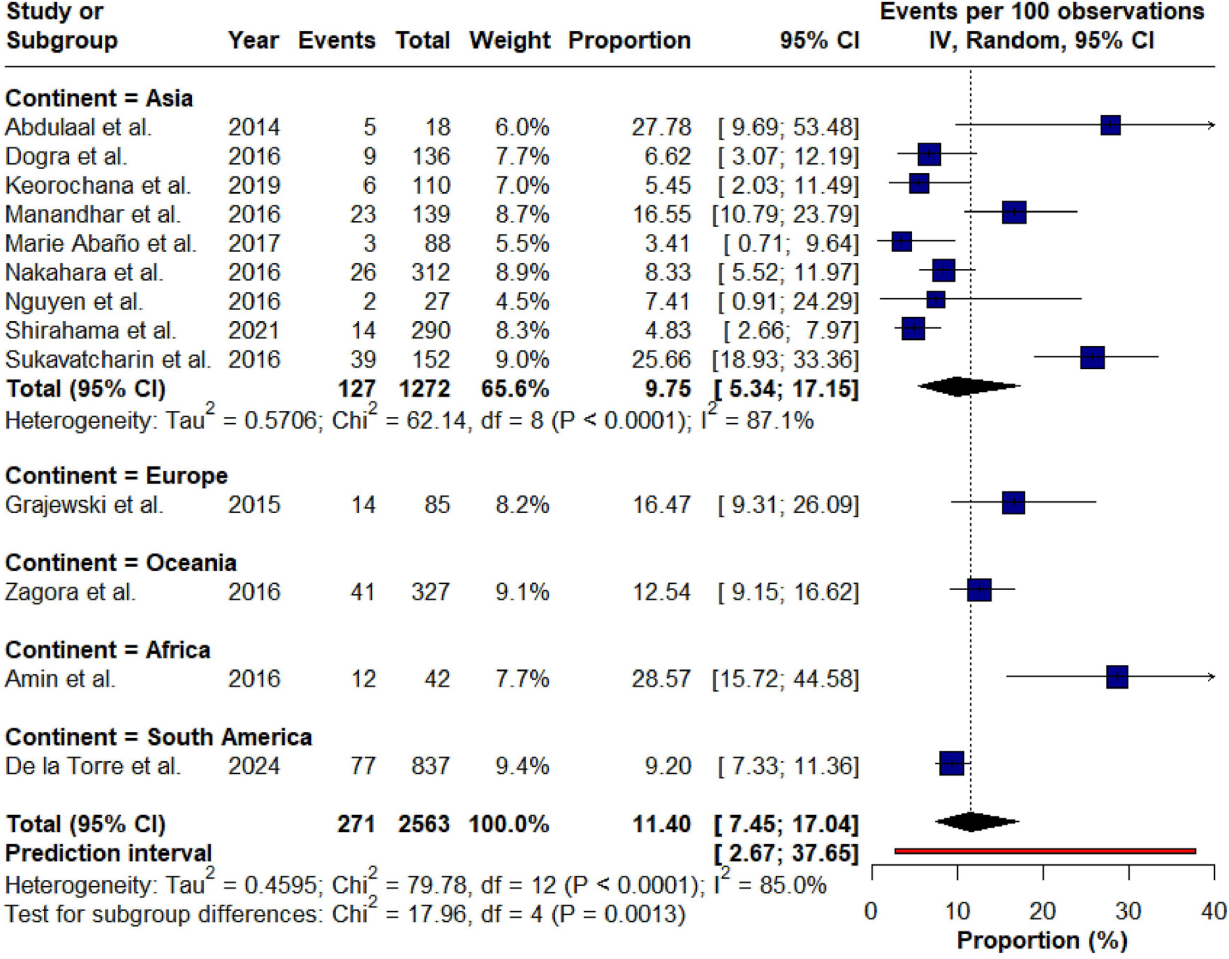
Continental subgroup analysis of herpetic uveitis proportion in the elderly population

**Fig. 3:**
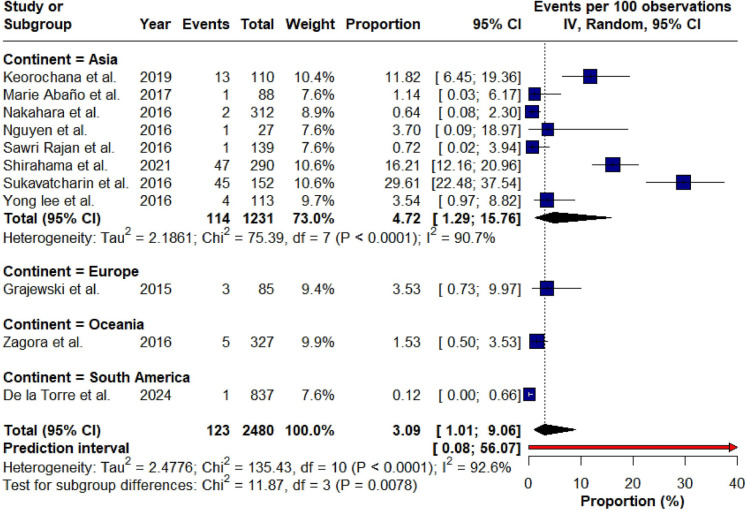
Continental subgroup analysis of cytomegalovirus uveitis proportion in the elderly population

**Fig. 4:**
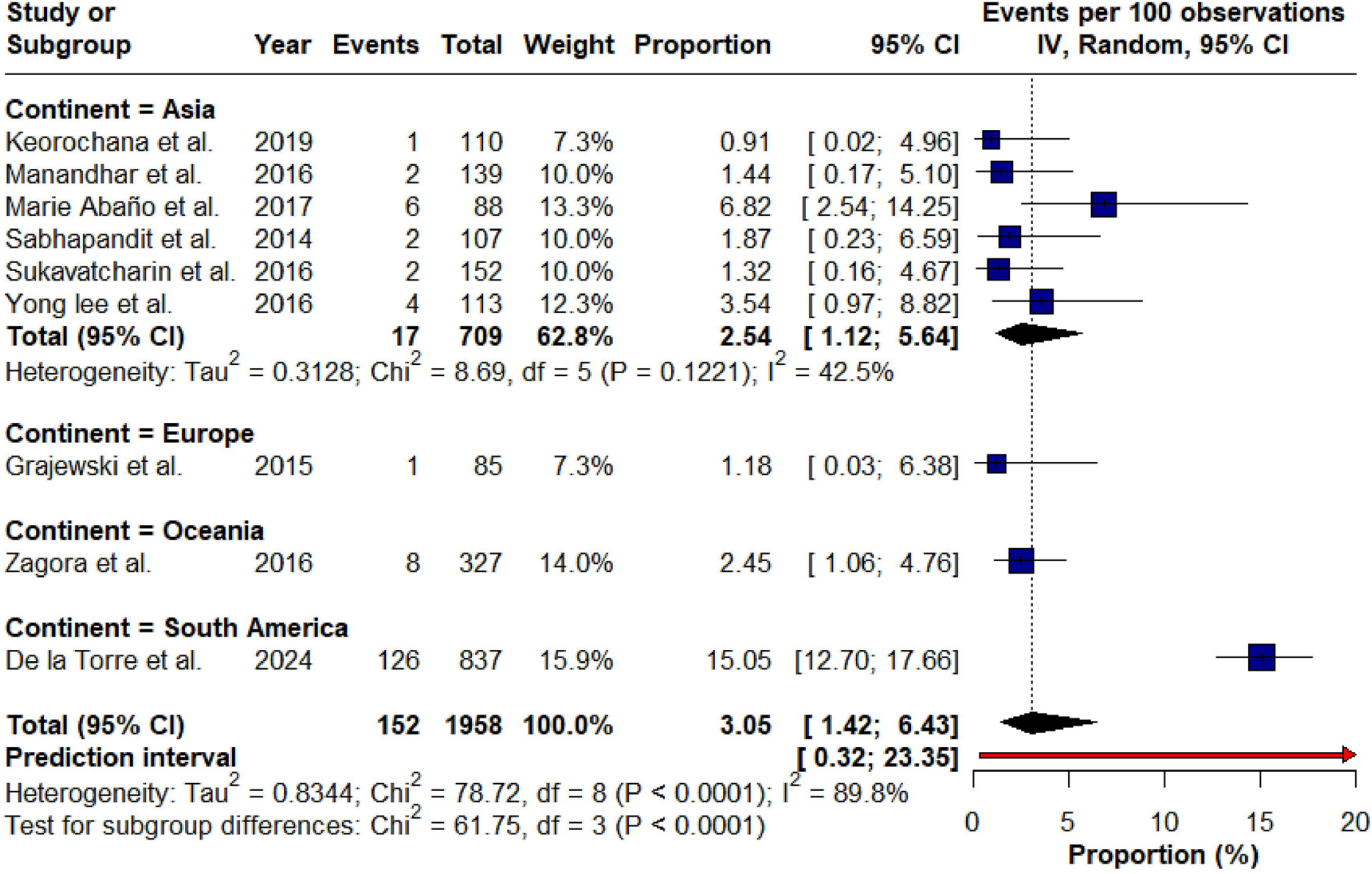
Continental subgroup analysis of toxoplasmosis uveitis proportion in the elderly population

**Fig. 5:**
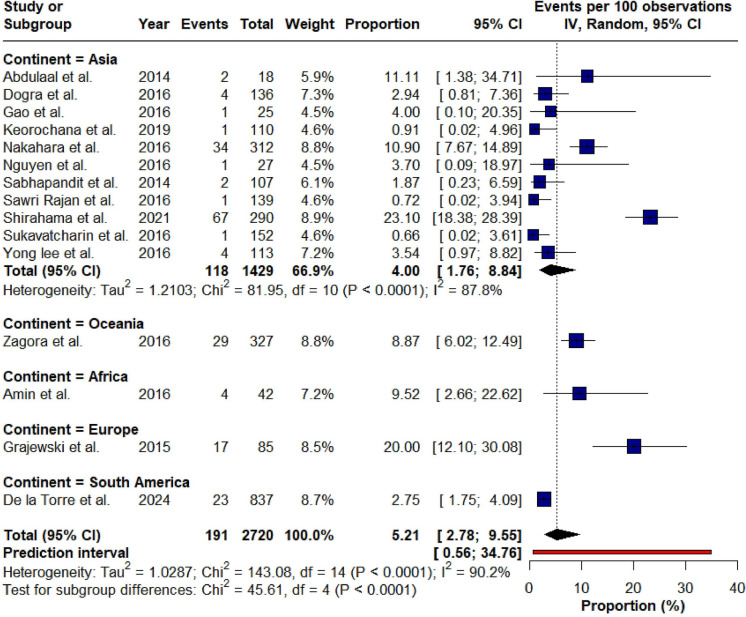
Continental subgroup analysis of sarcoidosis proportion in the elderly population

**Fig. 6:**
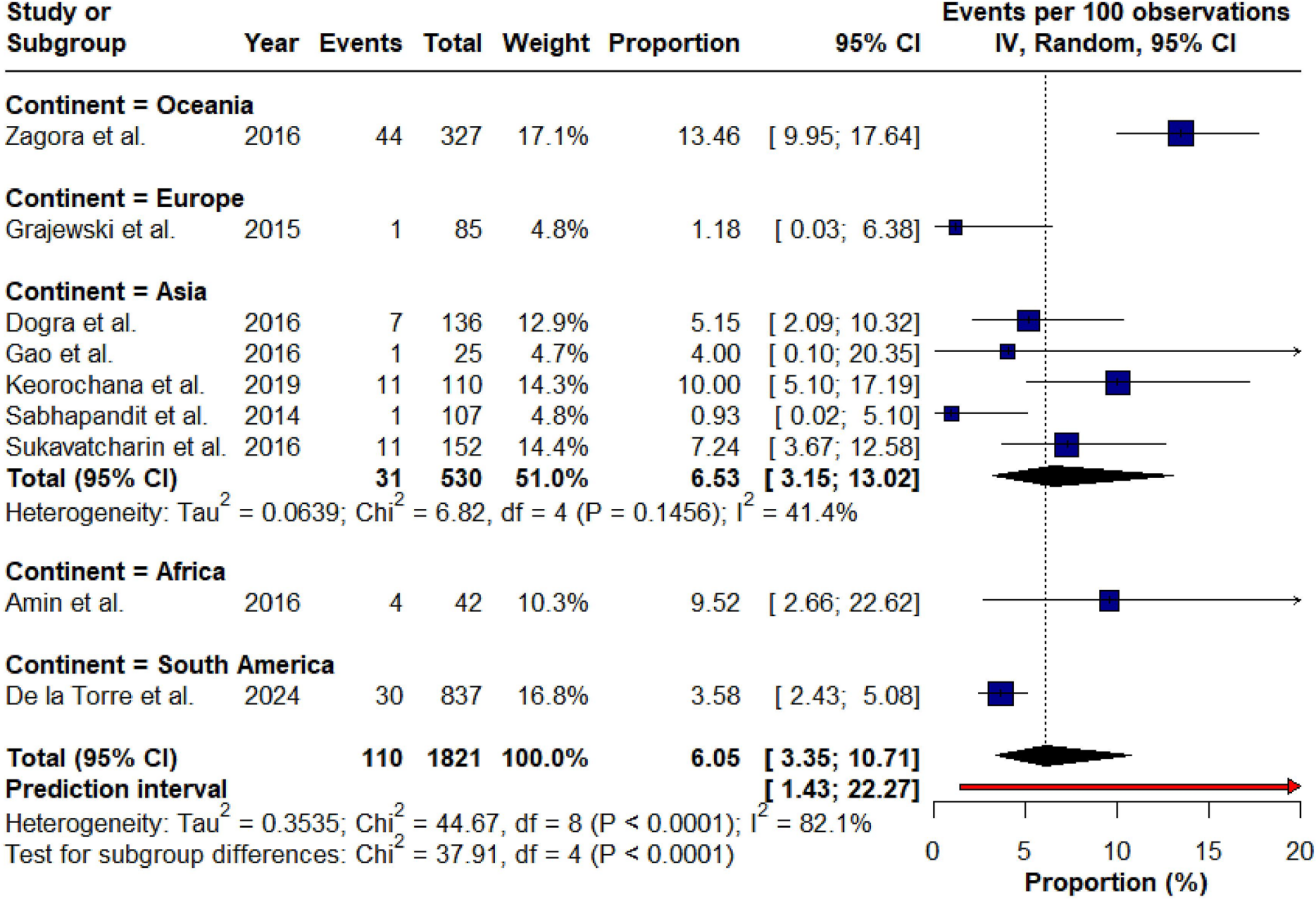
Continental subgroup analysis of HLA-B27-associated uveitis proportion in the elderly population

Figures 2, 3, 4, 5, 6 present the etiologic proportions by continent. Because only Asia contributed multiple studies (12 of 16), continental pooling was meaningful only for Asia; Europe, Africa, Oceania, and South America were each represented by a single study, so their values reflect individual studies rather than pooled continental estimates and were not compared statistically between continents. For herpetic uveitis, the pooled proportion within Asia was 9.75% (95% CI 5.34–17.15%), with residual heterogeneity remaining high (I2 = 87.1%). The corresponding single-study proportions were 28.57% (Africa), 16.47% (Europe), 12.54% (Oceania), and 9.20% (South America).

For sarcoidosis, the pooled proportion within Asia was 4.00% (95% CI 1.76–8.84%), while the single-study proportions were 20.00% (Europe), 9.52% (Africa), 8.87% (Oceania), and 2.75% (South America). For toxoplasmosis, the within-Asia pooled proportion was 2.54% (95% CI 1.12–5.64%), and subgrouping within Asia reduced heterogeneity substantially (I^2^ from 89.8 to 42.5%); the single South American study reported a higher proportion (15.05%). For HLA-B27, restricting to Asia reduced within-region heterogeneity (I^2^ from 82.1 to 41.4%), and the single Oceanian study reported a proportion of 13.46%. Within-Asia subgroup results for CMV, TB, VKH, lymphoma, and sympathetic ophthalmia are provided in Fig. 3 and Supplementary Figs. 13, 18, 19, 20. No formal between-continent significance testing was performed, because Asia was the only continent contributing more than one study.

Subgroup analyses were also performed for idiopathic uveitis (overall I^2^ = 91.4%) and endophthalmitis (overall I^2^ = 85.2%); because Asia was again the only continent with multiple studies, these are presented as within-Asia estimates alongside the single-study values for the other continents in Supplementary Figs. 16 and 17.

Sensitivity analyses restricted to studies applying standardized diagnostic criteria showed that for VKH disease, excluding the single study without explicit revised criteria (Grajewski et al.), the pooled proportion was 3.53% (95% CI 1.76–6.97%) with I^2^ = 83.3%, compared to 3.36% (95% CI 1.73–6.43%) with I2 = 82.5% for all 15 studies. For Behçet’s disease, restricting to studies using standard criteria (ISG or equivalent) yielded a pooled proportion of 1.35% (95% CI 0.43–4.15%) with I^2^ = 71.9%, compared to 1.56% (95% CI 0.55–4.36%) with I^2^ = 69.2% for all 10 studies (Figs. 14 and 15).

Leave-one-out analysis excluding de la Torre et al. (the largest study, N = 837) did not substantially alter any pooled estimate, with the largest shifts observed for CMV (3.09–4.15%) and TB (5.10–6.10%), confirming that no single study unduly influenced the overall findings.

**Fig. 7:**
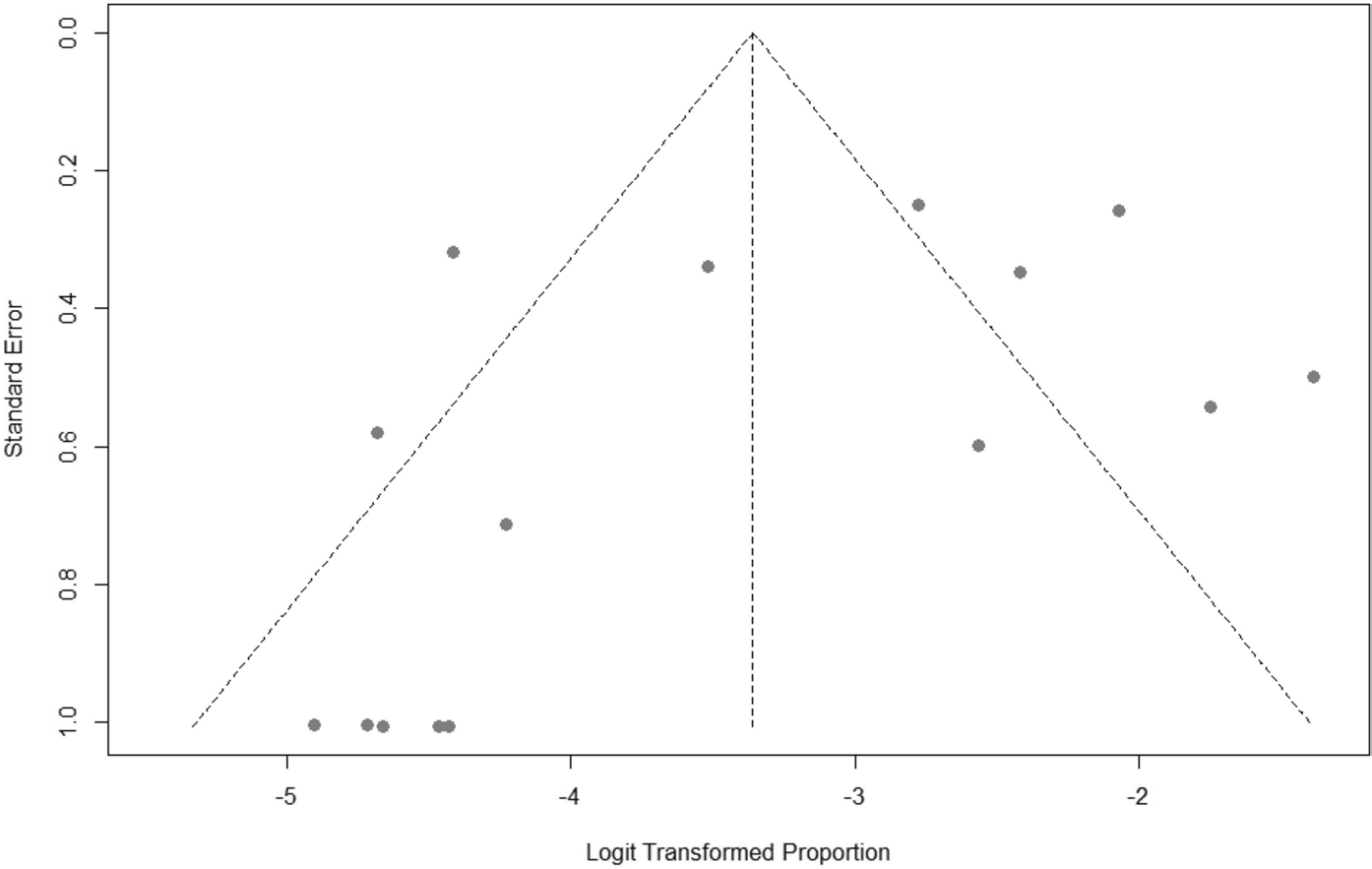
Funnel plot for assessment of publication bias in studies reporting Vogt-Koyanagi-Harada disease in the elderly population

Funnel plot analyses and Egger’s regression test were performed for the seven etiologies with 10 or more contributing studies. For VKH disease (k = 15), the funnel plot showed mild asymmetry, but Egger’s test was not significant (p = 0.131). Similarly, no significant asymmetry was detected for herpetic uveitis (p = 0.907), idiopathic uveitis (p = 0.875), or TB (p = 0.071). However, CMV showed significant funnel plot asymmetry (Egger’s p = 0.0005), suggesting possible publication bias or small-study effects for this etiology. Sarcoidosis also showed borderline significant asymmetry (p = 0.028). Behçet’s disease also showed significant asymmetry (Egger’s p = 0.036), potentially reflecting small-study effects or the inclusion of zero-event studies. The VKH funnel plot is presented in Fig. 7, while additional funnel plots are available in the Supplementary Material (Figs. 8, 9, 10, 11, 12, 21).

Risk of bias and methodological quality were assessed for all included studies using the Newcastle–Ottawa Scale and JBI checklist. As shown in Tables 3 and 4, all studies were rated as moderate to high quality, and no studies were excluded based on quality concerns.

This funnel plot checks for possible publication bias in studies looking at VKH disease. Each dot is a study. The bottom axis shows the result from each study, and the side axis shows how precise it is. If the dots are spread evenly around the middle line, there’s likely no bias. If they’re uneven or outside the funnel shape, bias might be present.

## Discussion

This systematic review and meta-analysis provide a comprehensive epidemiological characterization of uveitis in the elderly population. Idiopathic uveitis predominated with a pooled proportion of 41.45%. Herpetic uveitis was the leading infectious cause worldwide at 11.40% and 9.75% in Asia, whereas sarcoidosis topped non-infectious etiologies at 5.21% overall and 4.00% in Asia.

Herpetic uveitis was the most common infectious etiology, with an overall pooled proportion of 11.40% (95% CI 7.45–17.04%). In Asia, the proportion was slightly lower at 9.75% (95% CI 5.34–17.15%). This aligns with findings by Magesan et al., who identified herpes zoster ophthalmicus and herpes simplex virus as major causes of uveitis in the elderly, often triggered by immune senescence and viral reactivation [31].

Tuberculosis-related uveitis showed a pooled proportion of 5.10% (95% CI 2.58–9.83%) and remains especially relevant in TB-endemic regions. In India, where tuberculosis poses ongoing public health challenges, Magesan et al. identified it as a leading infectious cause of uveitis in older adults [31].

CMV-related uveitis had an overall pooled proportion of 3.09% (95% CI 1.01–9.06%), and within Asia, the only continent with poolable data, the proportion was 4.72% (95% CI 1.29–15.76%). The single South American study reported a low CMV proportion (0.12%); because the non-Asian continents were each represented by a single study, no between-continent comparison was made. Variation in CMV-related uveitis may reflect differences in access to PCR testing, which is key for early diagnosis. Limited availability in some regions can lead to underreporting [32–34]. Notably, Egger’s test showed significant funnel plot asymmetry for CMV (p = 0.0005), suggesting possible publication bias or small-study effects.

Sarcoidosis was the most common non-infectious etiology in our analysis, with an overall pooled proportion of 5.21% (95% CI 2.78–9.55%). Within Asia, the pooled proportion was 4.00% (95% CI 1.76–8.84%); the single European study (Grajewski et al.) reported a higher proportion of 20.00% (95% CI 12.81–29.84%), which, as a single-study value, cannot be interpreted as a continental estimate. Sarcoidosis is a major cause of non-infectious uveitis, particularly in Japan, where over 70% of cases involve ocular symptoms [35]. Hsu et al. highlighted its high burden in the Asia–Pacific region [36]. This may be linked to genetic factors like the HLA-DRB1*04-DQB1*0301 haplotype and environmental exposures at higher latitudes [37, 38].

In our study, autoimmune-related causes, such as HLA-B27-associated uveitis and Behçet’s disease (BD), were less common in the elderly population. HLA-B27-associated uveitis had a pooled proportion of 6.05% (95% CI 3.35–10.71%), reflecting a decline compared to younger cohorts. This aligns with findings by Chatzistefanou et al., who highlighted a decreased prevalence of HLA-B27-associated conditions in older adults, attributed to reduced immune hyperactivity and fewer autoimmune triggers with advancing age [39].

Similarly, Behçet’s disease-related uveitis had a pooled proportion of 1.35% (95% CI 0.43–4.15%) in the sensitivity analysis limited to studies that applied the International Study Group criteria [17]. The relatively low proportion of BD-related uveitis in our cohort may also reflect the advanced age of the study population, as BD is typically diagnosed in younger adults [40–42].

Idiopathic uveitis emerged as the most prevalent category, with a pooled proportion of 41.45% (95% CI 30.63–53.16%). Previous studies similarly reported rates between 34% and 46.9% [43, 44]. Grumet et al. [45] and Magesan et al. [31] attributed this high proportion to age-related immune changes, overlapping comorbidities, and subtle clinical presentations. Notably, Choi et al. found that 29% of initially undifferentiated cases were later reclassified following specialized evaluation [46]. A single study (de la Torre et al.) reported 50 of 837 elderly patients (5.97%) as undetermined, defined as cases with incomplete evaluation. This was not pooled due to the single-study estimate.

This systematic review and meta-analysis have several limitations that may influence the interpretation of findings. First, heterogeneity was high across most etiologies, which limits the precision of the pooled estimates. Sensitivity analyses restricted to studies applying standardized diagnostic criteria (International Study Group criteria for Behçet disease and the revised criteria for Vogt–Koyanagi–Harada disease [17, 18]) did not meaningfully reduce heterogeneity, indicating that differences in diagnostic frameworks are not its primary source; subgrouping by continent attenuated heterogeneity for some etiologies but not others, as reported in the Results. Second, the geographic evidence base was markedly unbalanced: only Asia contributed multiple studies (12 of 16), whereas Europe, Africa, Oceania, and South America were each represented by a single study. The proportions reported for these continents therefore reflect individual cohorts and should not be read as continental estimates or used for between-continent comparison. Third, nearly all included studies were retrospective, which carries the selection and ascertainment biases inherent to that design. Fourth, formal testing indicated possible publication bias or small-study effects for several etiologies, with significant Egger asymmetry for CMV (p = 0.0005), sarcoidosis (p = 0.028), and Behçet disease (p = 0.036); the decision not to search gray literature, taken to preserve scope and consistency, may have contributed to this. Fifth, the analysis relied on aggregate counts reported in the published papers rather than patient-level data; this dependence on summary figures was the source of the extraction discrepancies identified and corrected during revision and limits the granularity of any reanalysis. In addition, few studies reported endophthalmitis as an etiology of uveitis; although it is a distinct intraocular infection, we retained it to reflect the original authors’ classifications and ensure consistency. Finally, the focus on tertiary referral centers may have overrepresented severe or refractory cases, underestimating the proportion of milder forms of uveitis often managed in community settings.

In conclusion, this meta-analysis characterizes the etiological landscape of uveitis in elderly patients seen at tertiary referral centers. Herpetic uveitis is the leading infectious etiology and sarcoidosis is the principal non-infectious etiology. Continental subgroup analyses showed that geographic factors partly explain the observed heterogeneity for toxoplasmosis (within-Asia I2 dropping to 42.5%) and HLA-B27 (within-Asia I2 dropping to 41.4%), though heterogeneity remained high within subgroups for most other etiologies. These findings represent patterns within tertiary referral cohorts rather than precise population-level estimates, and should be interpreted in that context. Standardized diagnostic reporting and additional population-based research, particularly from underrepresented regions, are essential to refine these estimates and guide age-tailored management.

## Supplementary Information

Below is the link to the electronic supplementary material.

Supplementary file1 (DOCX 39 KB)

Supplementary file2 (DOCX 38 KB)

Supplementary file3 (DOCX 15 KB)

Supplementary file4 (DOCX 409 KB)

## Data Availability

No datasets were generated or analysed during the current study.
