# Supplementary material for: Epidemiological characterization of uveitis in the elderly population: a systematic review and meta-analysis
Source: Int Ophthalmol. 2026 Jul 19;46(1):300. doi: 10.1007/s10792-026-04170-z (PMC13381384; doi:10.1007/s10792-026-04170-z)
Supplement: Supplementary file 1 — Supplementary file1 (DOCX 39 KB) [file 10792_2026_4170_MOESM1_ESM.docx]

**MOOSE (Meta-analyses Of Observational Studies in Epidemiology) Checklist**

*Epidemiological Characterization of Uveitis in Elderly Population:
A Systematic Review and Meta-Analysis*

*A reporting checklist for Authors, Editors, and Reviewers of Meta-analyses of Observational Studies. You must report the page number in your manuscript where you consider each of the items listed in this checklist. If you have not included this information, either revise your manuscript accordingly before submitting or note N/A.*

| **Reporting Criteria** | **Reported (Yes/No)** | **Reported on Page No.** |
| --- | --- | --- |
| **Reporting of Background** |  |  |
| Problem definition | Yes | p. 3 (Introduction): Uveitis as a significant cause of vision loss in the elderly; differing common diagnoses across studies; aging global population increases relevance |
| Hypothesis statement | Yes | pp. 2–3 (Abstract and Introduction): Aims to characterize etiological patterns of uveitis in individuals aged 60 years or older |
| Description of Study Outcome(s) | Yes | pp. 2, 6–7 (Abstract and Methods – Statistical Analyses): Proportion of each uveitis etiology among elderly uveitis cases, with 95% CIs |
| Type of exposure or intervention used | N/A | N/A — this is a descriptive epidemiological study characterizing disease etiology proportions; no exposure or intervention was assessed |
| Type of study design used | Yes | pp. 3–4 (Methods): Systematic review and meta-analysis of cross-sectional and cohort studies |
| Study population | Yes | pp. 2, 4 (Abstract and Inclusion Criteria): Patients aged 60 years or older with uveitis |
| **Reporting of Search Strategy** |  |  |
| Qualifications of searchers (e.g., librarians and investigators) | Yes | pp. 4–5 (Data Extraction): Three independent reviewers (T.L, A.G, J.M) — authors/medical researchers who conducted all screening and extraction |
| Search strategy, including time period included in the synthesis and keywords | Yes | pp. 3–4 (Search Strategy): PubMed/MEDLINE, Scopus, PMC, Web of Science; search terms listed; January 1, 2005 to January 1, 2025; full strategies in Supplementary Material |
| Effort to include all available studies, including contact with authors | Partial | p. 4 (Search Strategy): Relevant articles were cross-referenced for additional manuscripts. No language restrictions applied. Contact with authors not reported |
| Databases and registries searched | Yes | pp. 3–4 (Search Strategy): PubMed/MEDLINE, Scopus, PubMed Central (PMC), and Web of Science |
| Search software used, name and version, including special features used (e.g., explosion) | No | Not explicitly stated — databases were searched directly; no specific search software reported |
| Use of hand searching (e.g., reference lists of obtained articles) | Yes | p. 4 (Search Strategy): "Relevant articles were cross-referenced for additional manuscripts that were not directly found through the above search" |
| List of citations located and those excluded, including justification | Yes | p. 8 (Results): 10,829 citations identified; 4,563 duplicates removed; 6,266 screened; 785 full-text evaluated; 16 included. PRISMA flow diagram (Figure 1) details exclusion reasons |
| Method for addressing articles published in languages other than English | Yes | p. 4 (Search Strategy): "No language restrictions were applied" |
| Method of handling abstracts and unpublished studies | Yes | pp. 11–12 (Limitations): "We did not use gray literature to keep the scope focused and ensure consistency across studies, though this may introduce some publication bias" |
| Description of any contact with authors | No | Not reported — no description of author contact for data clarification or additional information |
| **Reporting of Methods** |  |  |
| Description of relevance or appropriateness of studies assembled for assessing the hypothesis to be tested | Yes | p. 4 (Inclusion/Exclusion Criteria): Studies required to include patients aged ≥60, cross-sectional or cohort design, known etiology of uveitis, published 2005–2025 |
| Rationale for the selection and coding of data (e.g., sound clinical principles or convenience) | Yes | pp. 4–5 (Data Extraction): Definitions of "idiopathic" vs. "undetermined" based on clinical principles; reclassification of ambiguous terms after methods review; grouping of idiopathic subtypes |
| Documentation of how data were classified and coded (e.g., multiple raters, blinding, and interrater reliability) | Yes | pp. 4–5 (Data Extraction): Three independent reviewers with fourth reviewer for disagreements; two independent reviewers for quality assessment with third for disagreements |
| Assessment of confounding (e.g., comparability of cases and controls in studies where appropriate) | N/A | N/A — descriptive prevalence study without between-group comparisons; the JBI checklist was used instead of the NOS comparability domain for cross-sectional studies (p. 6) |
| Assessment of study quality, including blinding of quality assessors; stratification or regression on possible predictors of study results | Yes | pp. 5–6 (Risk of Bias Assessment): Newcastle–Ottawa Scale for cohort studies; JBI Critical Appraisal Checklist for cross-sectional studies; two independent reviewers with third for disagreements. Results in Tables 3–4 (p. 9) |
| Assessment of heterogeneity | Yes | pp. 6–7 (Statistical Analyses): Cochran’s Q test and I² statistic; I² > 50% = substantial heterogeneity; continental subgroup analyses for I² > 75%; sensitivity analyses for diagnostic criteria |
| Description of statistical methods (e.g., complete description of fixed or random effects models, justification of whether the chosen models account for predictors of study results, dose-response models, or cumulative meta-analysis) in sufficient detail to be replicated | Yes | pp. 6–7 (Statistical Analyses): Random-effects model with REML estimator; logit transformation; Hartung–Knapp adjustment; pooling when k ≥ 2; funnel plots and Egger’s test for k > 10; continental subgroups; leave-one-out sensitivity; R 4.4.2 with meta package 8.3-0 |
| Provision of appropriate tables and graphics | Yes | Throughout Results (pp. 8–9): Table 1 (study characteristics), Table 2 (meta-analysis results), Tables 3–4 (quality assessment); forest plots (Figures 2–7), funnel plots (Figures 7–12), subgroup plots (Figures 2–6), sensitivity plots (Figures 14–15) |
| **Reporting of Results** |  |  |
| Table giving descriptive information for each study included | Yes | p. 8; Table 1: First author, year, study period, country, study type, sample size, and etiology data for all 16 studies |
| Results of sensitivity testing (e.g., subgroup analysis) | Yes | pp. 8–9 (Results): Continental subgroup analyses (pp. 8–9), leave-one-out excluding de-la-Torre et al. (p. 9), Behçet and VKH sensitivity restricted to standardized criteria (p. 9; Figures 14–15) |
| Indication of statistical uncertainty of findings | Yes | pp. 8–9 (Results): 95% CIs for all pooled proportions; I² and p-values for heterogeneity; Egger’s test p-values for publication bias |
| **Reporting of Discussion** |  |  |
| Quantitative assessment of bias (e.g., publication bias) | Yes | p. 9 (Results): Funnel plots and Egger’s regression test for 7 etiologies with k ≥ 10; CMV significant asymmetry (p = 0.0005), Behçet (p = 0.036), sarcoidosis borderline (p = 0.028). Figure 7; Supplementary Figures 8–12 |
| Justification for exclusion (e.g., exclusion of non-English-language citations) | Yes | pp. 4, 11–12: Inclusion/exclusion criteria stated (p. 4); no language restrictions (p. 4); gray literature excluded with justification (pp. 11–12) |
| Assessment of quality of included studies | Yes | p. 9 (Results): All studies rated moderate to high quality; no studies excluded based on quality. Tables 3–4 |
| **Reporting of Conclusions** |  |  |
| Consideration of alternative explanations for observed results | Yes | pp. 10–12 (Discussion): Geographic variation, immune senescence, differences in diagnostic access (e.g., PCR for CMV), genetic factors (HLA haplotypes for sarcoidosis), age-related decline in autoimmune conditions, tertiary referral bias |
| Generalization of the conclusions (i.e., appropriate for the data presented and within the domain of the literature review) | Yes | pp. 11–12 (Limitations and Conclusion): Findings represent tertiary referral cohort patterns, not precise population-level estimates; Asian-weighted sample limits generalizability; absence of North American studies noted |
| Guidelines for future research | Yes | p. 12 (Conclusion): Standardized diagnostic reporting and additional population-based research, particularly from underrepresented regions, are recommended |
| Disclosure of funding source | Yes | p. 12 (Financial Disclosure): "The authors did not report any sources of funding support" |

*Stroup DF, Berlin JA, Morton SC, et al. Meta-analysis of observational studies in epidemiology: a proposal for reporting. JAMA 2000;283(15):2008-2012.*
