# Supplementary material for: Epidemiological characterization of uveitis in the elderly population: a systematic review and meta-analysis
Source: Int Ophthalmol. 2026 Jul 19;46(1):300. doi: 10.1007/s10792-026-04170-z (PMC13381384; doi:10.1007/s10792-026-04170-z)
Supplement: Supplementary file 3 — Supplementary file3 (DOCX 15 KB) [file 10792_2026_4170_MOESM3_ESM.docx]

*Scopus Search Strategy*

*TITLE-ABS-KEY(uveit* OR "intraocular inflammation" OR "ocular inflammation") AND TITLE-ABS-KEY(epidemiolog* OR etiolog* OR "clinical patterns" OR "clinical features" OR prevalence OR incidence OR* “Patterns of”*) AND TITLE-ABS-KEY(“Elderly” OR “Geriatric” OR “Aged” OR "Older Adults")*

3,670 Results

*PubMed Search Strategy*

(Uveit*[tiab] OR "Intraocular inflammation"[tiab] OR "Ocular inflammation"[tiab]) AND (Uveitis/epidemiology[MeSH] OR Epidemiolog*[tiab] OR Etiolog*[tiab] OR "Clinical Patterns"[tiab] OR "Clinical features"[tiab] OR Prevalence[tiab] OR Incidence[tiab] OR “Patterns of” [tiab]) AND (Elderly[MeSH] OR Elderly[tiab] OR Geriatric[MeSH] OR Geriatric[tiab] OR Aged[MeSH] OR Aged[tiab] OR “Older Adults”[MeSH] OR “Older Adults”[tiab])

1,065 Results

*PubMed Central Search Strategy*

(Uveitis[MeSH Terms] OR Uveiti*[Title/Abstract] OR "intraocular inflammation"[Title/Abstract] OR "ocular inflammation"[Title/Abstract] ) AND ( Epidemiology[MeSH Terms] OR Etiology[MeSH Terms] OR Epidemiology[*All Fields*] OR Etiology[*All Fields*] OR Incidence[*All Fields*] OR Prevalence[*All Fields*] OR "clinical features"[*All Fields*] OR "Clinical Patterns"[*All Fields*] OR “Patterns of”) AND (Elderly[All Fields] OR Geriatric[All Fields] OR Aged[All Fields] OR "Older Adults"[All Fields])

1,790 Results

*Web of science Search Strategy*

*TS=("uveit*" OR "intraocular inflammation" OR "ocular inflammation") AND TS=("epidemiolog*" OR "etiolog*" OR "clinical patterns" OR "clinical features" OR "prevalence" OR "incidence" OR* “Patterns of”*) AND TS=(“Elderly” OR “Geriatric” OR “Aged” OR "Older Adults")*

4,301 Results
