# Supplementary material for: Epidemiological characterization of uveitis in the elderly population: a systematic review and meta-analysis
Source: Int Ophthalmol. 2026 Jul 19;46(1):300. doi: 10.1007/s10792-026-04170-z (PMC13381384; doi:10.1007/s10792-026-04170-z)

| Risk of bias:  Table 3. JBI cross-sectional: |  |  |  |  |  |  |  |  |
| --- | --- | --- | --- | --- | --- | --- | --- | --- |
| Study | (1) | (2) | (3) | (4) | (5) | (6) | (7) | (8) |
| De la Torre et al. [15] | Y | Y | Y | Y | Y | N | Y | Y |
| Shirahama et al. [7] | Y | Y | Y | Y | Y | Y | Y | Y |
| Keorochana et al. [16] | Y | Y | Y | Y | N | N | Y | Y |
| Abaño et al. [8] | Y | Y | Y | Y | Y | Y | Y | Y |
| Dogra et al. [17] | Y | Y | Y | Y | Y | Y | Y | Y |
| Sukavatcharin et al. [22] | Y | Y | Y | Y | Y | N | Y | Y |
| Lee et al. [23] | Y | Y | Y | Y | Y | N | Y | Y |
| Manandhar et al. [24] | Y | Y | Y | Y | N | N | Y | Y |
| Nakahara et al. [26] | Y | Y | N | Y | Y | Y | Y | N |
| Abdulaal et al. [6] | Y | Y | Y | Y | Y | Y | Y | Y |
|  |  |  |  |  |  |  |  |  |

N, No; U, Unclear; Y, Yes.
Q1. Were the criteria for inclusion in the sample clearly defined?, Q2. Were the study subjects and the setting described in detail?, Q3. Was the exposure measured in a valid and reliable way?, Q4. Were objective, standard criteria used for measurement of the condition?, Q5. Were confounding factors identified?, Q6. Were strategies to deal with confounding factors stated?, Q7. Were the outcomes measured in a valid and reliable way?, Q8. Was appropriate statistical analysis used?

Table 4. The Newcastle-Ottawa Scale (NOS) cohort:

|  |  |  |  |  |  |  |  |  |  |  |  |
| --- | --- | --- | --- | --- | --- | --- | --- | --- | --- | --- | --- |
| **References** | **Study type** | **Selection** | | | | **Comparability** | | **Exposure/Outcome** | | | **Total** |
|  |  | **1** | **2** | **3** | **4** | **1a** | **1b** | **1** | **2** | **3** |  |
| [Zagora et al. [18]](https://pubmed.ncbi.nlm.nih.gov/?term=Zagora+SL&cauthor_id=27901620) | Cohort | * | NA | * | * | * | * | * | * | NA | 7 |
| Amin et al. [19] | Cohort | * | NA | * | * | * | NA | * | * | NA | 6 |
| Sabhapandit et al. [20] | Cohort | * | NA | * | * | * | * | * | * | NA | 7 |
| Nguyen et al. [21] | Cohort | * | NA | * | * | * | * | * | * | NA | 7 |
| Gao et al. [25] | Cohort | * | NA | * | * | * | NA | * | * | NA | 6 |
| Grajewski et al. [27] | Cohort | * | NA | * | * | * | NA | * | * | NA | 6 |
|  |  |  |  |  |  |  |  |  |  |  |  |

Funnel plots:

Fig 8. Funnel plot for assessment of publication bias in studies reporting tuberculosis-associated uveitis in the elderly population


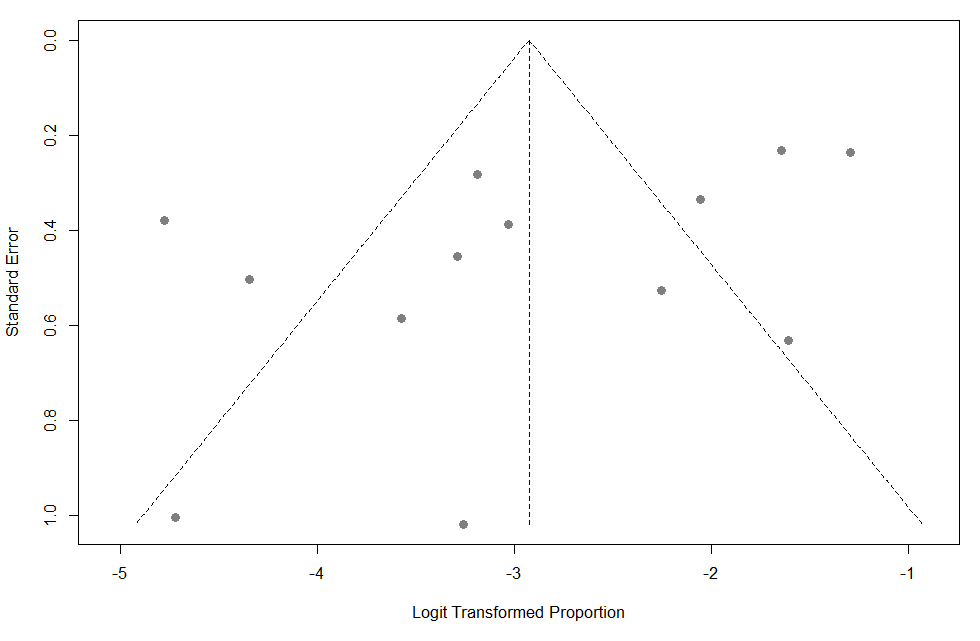


Fig 9. Funnel plot for assessment of publication bias in studies reporting herpetic uveitis in the elderly population


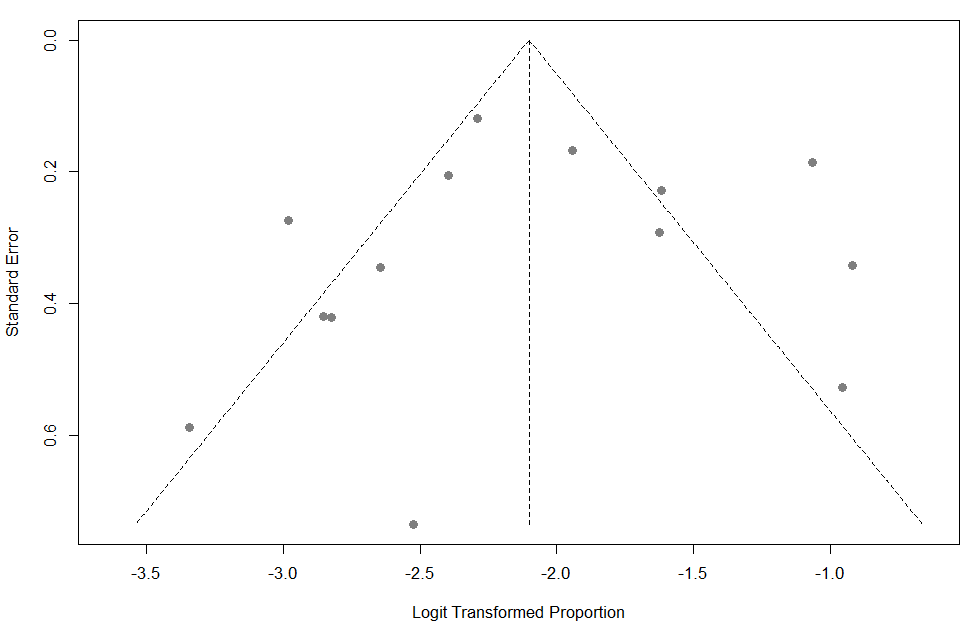


Fig 10. Funnel plot for assessment of publication bias in studies reporting cytomegalovirus uveitis in the elderly population


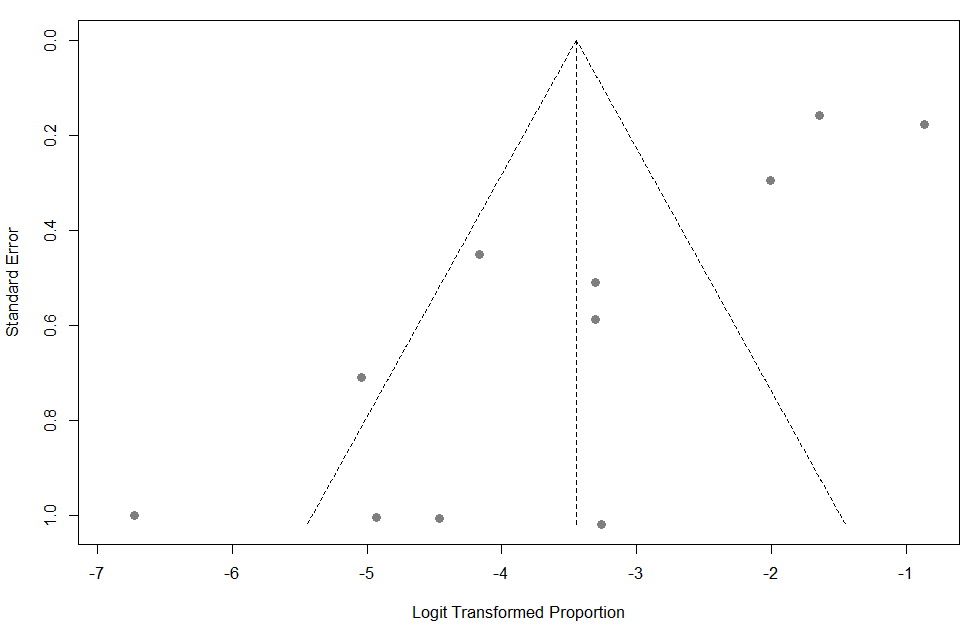


Fig 11. Funnel plot for assessment of publication bias in studies reporting sarcoidosis in the elderly population


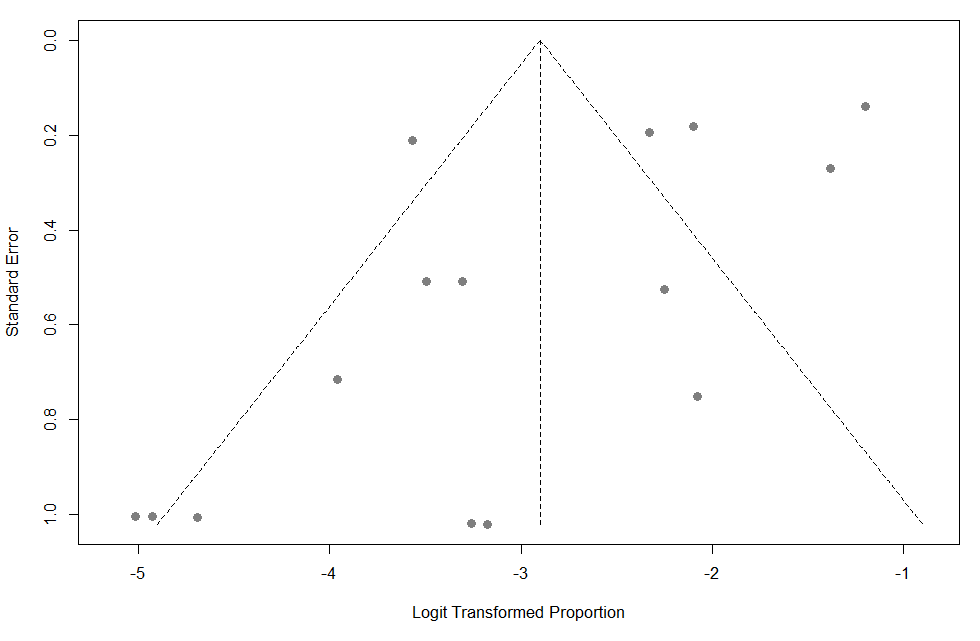


Fig 12. Funnel plot for assessment of publication bias in studies reporting idiopathic uveitis in the elderly population


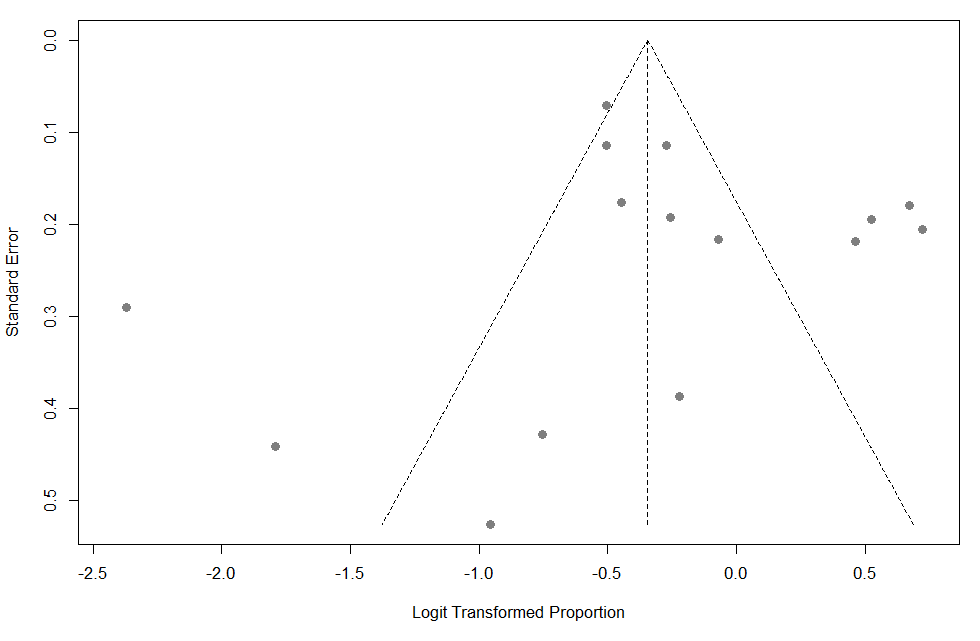


Continental subgroup analysis

Fig 13. Continental subgroup analysis of Vogt-Koyanagi-Harada disease proportion in the elderly population


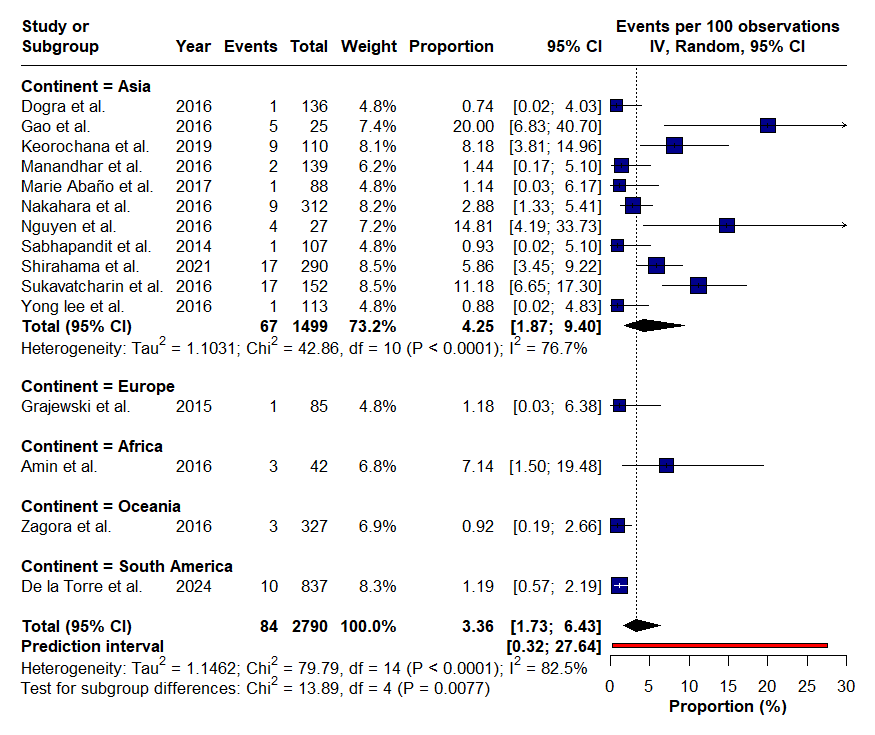


Sensitivity analysis based on diagnostic criteria

Fig 14. Sensitivity analysis of Behçet disease proportion restricted to studies applying International Study Group diagnostic criteria


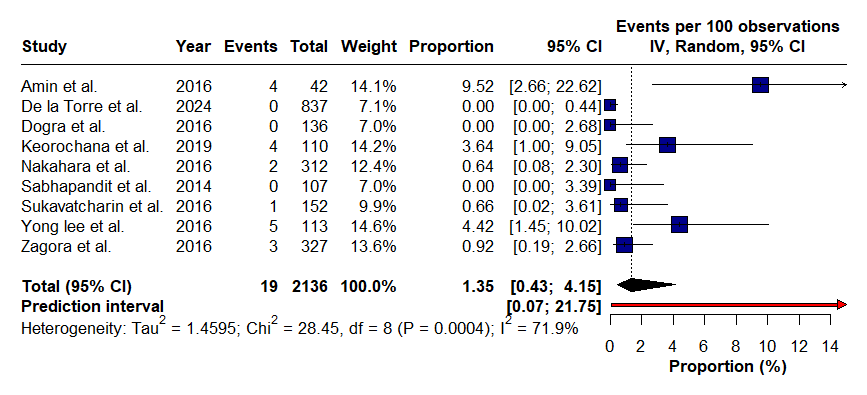


Fig 15. Sensitivity analysis of Vogt-Koyanagi-Harada disease proportion restricted to studies applying revised diagnostic criteria


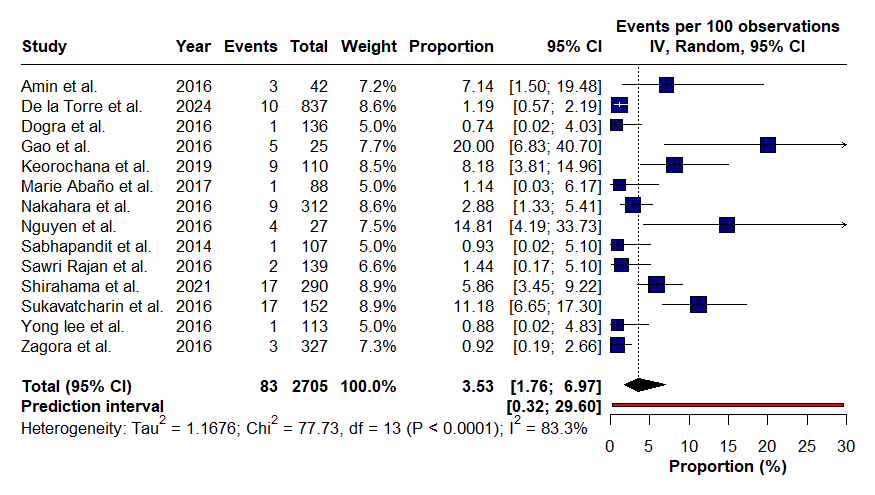


Fig 16. Continental subgroup analysis of endophthalmitis proportion in the elderly population


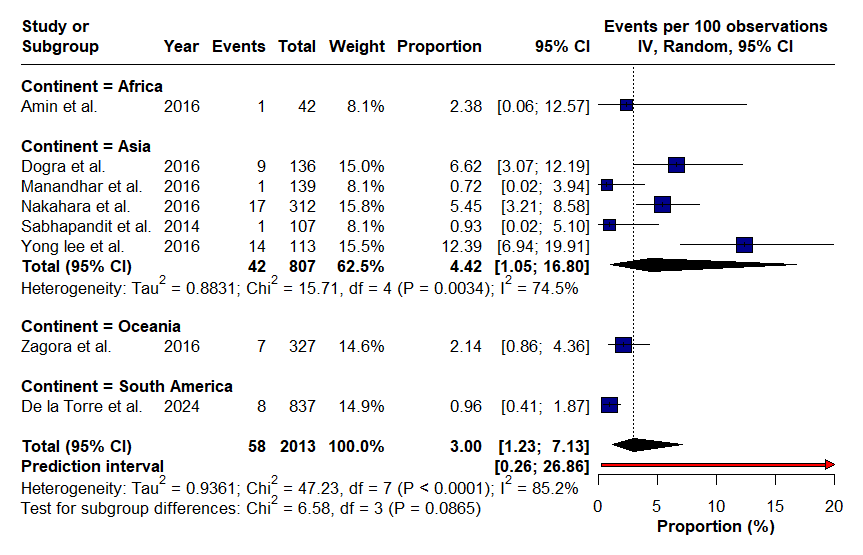


Fig 17. Continental subgroup analysis of idiopathic uveitis proportion in the elderly population


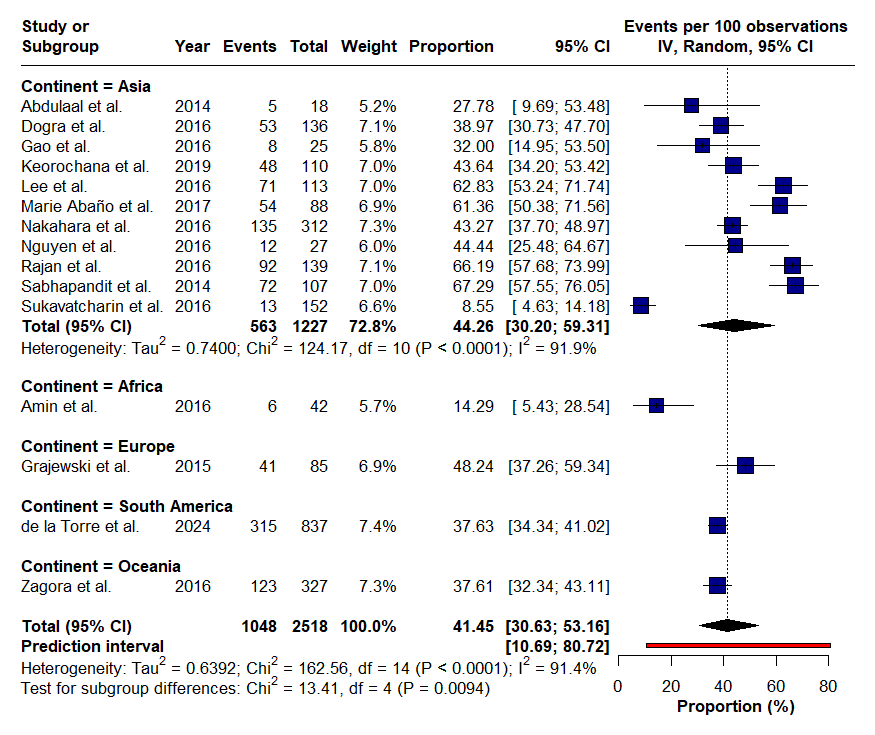


Fig 18. Continental subgroup analysis of lymphoma-associated uveitis proportion in the elderly population


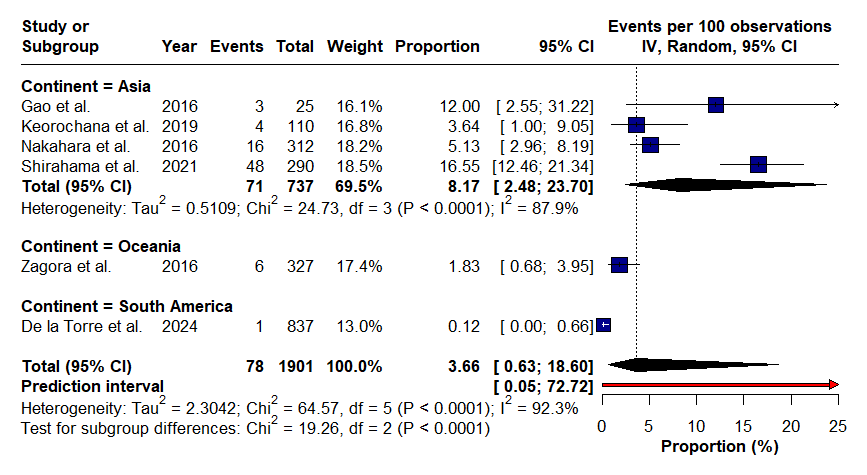


Fig 19. Continental subgroup analysis of sympathetic ophthalmia proportion in the elderly population


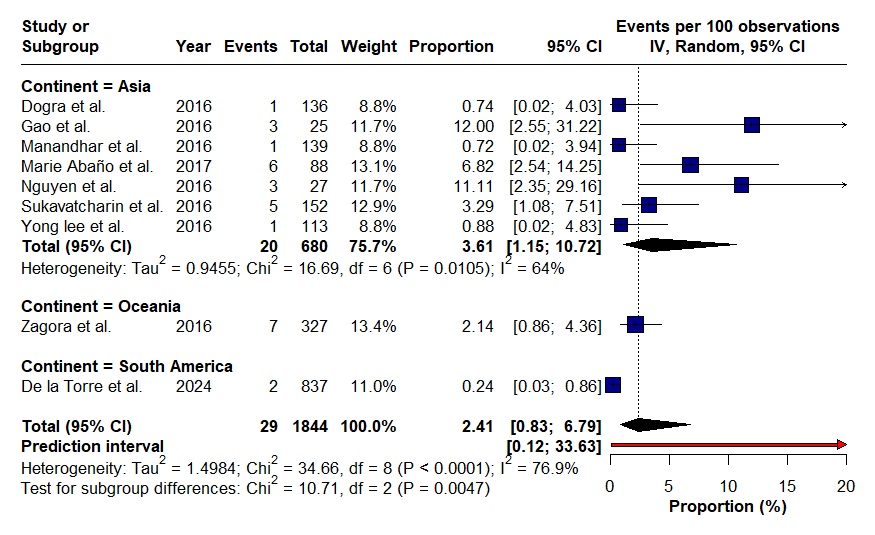


Fig 20. Continental subgroup analysis of tuberculosis-associated uveitis proportion in the elderly population
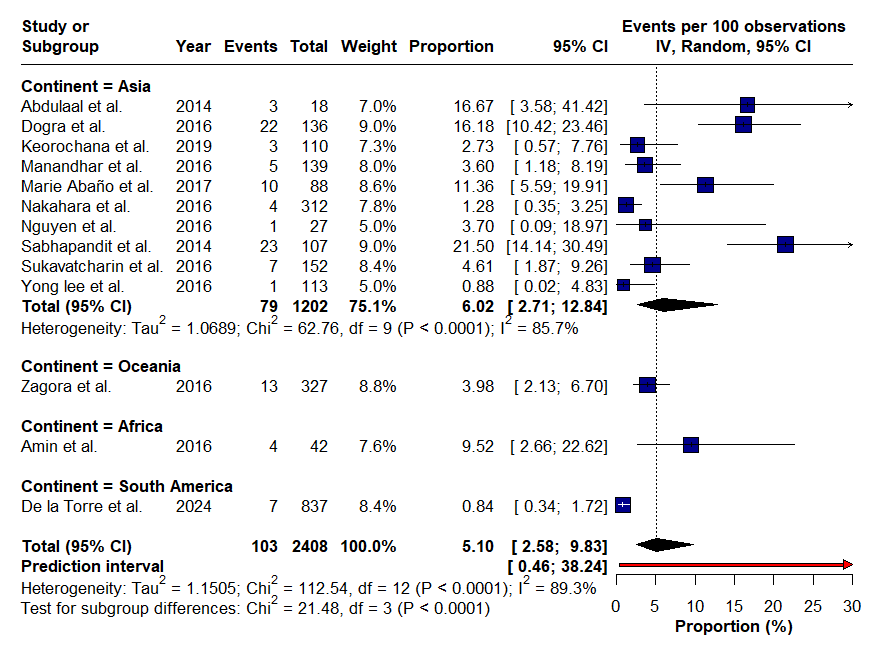


Fig 21. Funnel plot for assessment of publication bias in studies reporting Behçet disease in the elderly population


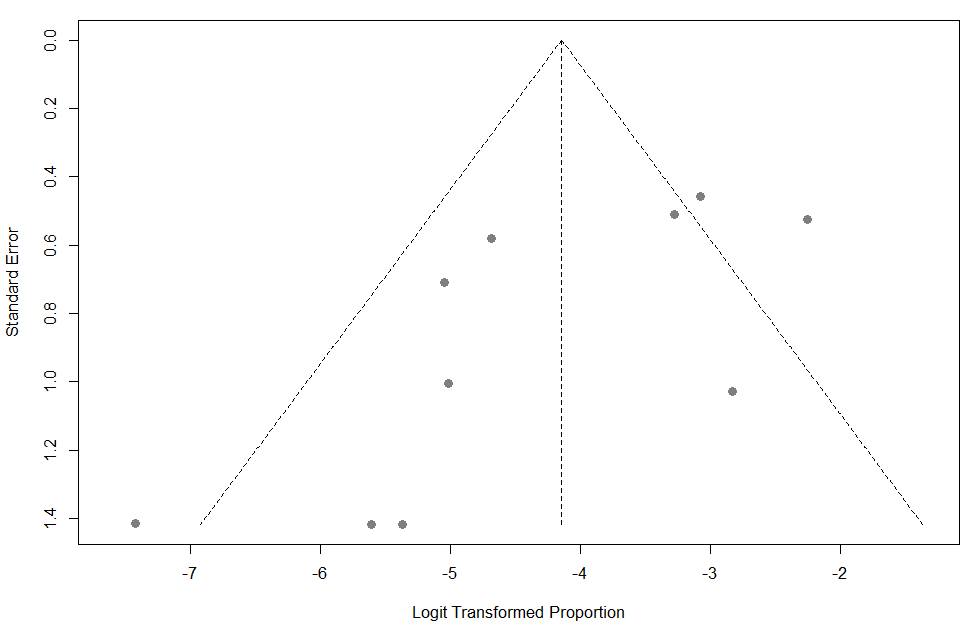

Supplement: Supplementary file 4 — Supplementary file4 (DOCX 409 KB) [file 10792_2026_4170_MOESM4_ESM.docx]
